# Antibacterial activity and mechanism of flavonoids from *Chimonanthus salicifolius* S. Y. Hu. and its transcriptome analysis against *Staphylococcus aureus*

**DOI:** 10.3389/fmicb.2022.1103476

**Published:** 2023-01-10

**Authors:** Huan Zhou, Lingli Chen, Kehui Ouyang, Qingfeng Zhang, Wenjun Wang

**Affiliations:** ^1^Jiangxi Key Laboratory of Natural Products and Functional Food, College of Food Science and Engineering, Jiangxi Agricultural University, Nanchang, China; ^2^College of Animal Science and Technology, Jiangxi Agricultural University, Nanchang, China

**Keywords:** flavonoids, *Chimonanthus salicifolius* S. Y. Hu., antibacterial activity, antibacterial mechanisms, *Staphylococcus aureus*, transcriptome analysis

## Abstract

**Introduction:**

*Chimonanthus salicifolius* S. Y. Hu. (FCS) possess many biological activities, but the antibacterial activity and underlying mechanisms of flavonoids from Chimonanthus salicifolius S. Y. Hu. (FCS) is still unknown.

**Method:**

Maximum diameter of inhibition zone (DIZ), maximum diameter of inhibition zone (DIZ), the lowest minimum inhibition concentration (MIC), and the lowest minimum bactericide concentration (MBC) were used to detect the antibacterial activity. Meanwhile, related enzyme activities, the transcriptome analysis and quantitative RT-PCR were used to investigate the antibacterial activity mechanisms.

**Results:**

The results showed that FCS (with a purity of 84.2 ± 2.0%) has potential effects on tested strains with the maximum diameter of inhibition zone (DIZ) was 15.93 ± 2.63 mm, the lowest minimum inhibition concentration (MIC) was 1.56 mg/ml and the lowest minimum bactericide concentration (MBC) was 6.25 mg/ml. In addition, the bacterial growth curve test, release of extracellular alkaline phosphatase (AKP), loss of intracellular components, DNA damage and transmission electron microscope (TEM) suggested that FCS could destroy the cell wall and membrane, cause the loss of intracellular substance, cause DNA damage and even lead to cell death. Moreover, the antibacterial mechanism of FCS against *Staphylococcus aureus* (*S. aureus*, Gram-positive bacteria) was further confirmed by the transcriptome analysis and quantitative RT-PCR at the molecular level for the first time. A total of 671 differentially expressed genes (DEGs) were identified after treated with FCS (1/2 MIC), with 338 and 333 genes showing up-regulation and down-regulation, respectively. The highlighted changes were those related to the biosynthesis of bacteria wall and membrane, DNA replication and repair, and energy metabolism.

**Discussion:**

Overall, our research provides theoretical guidance for the application of FCS, which is expected to be potentially used as a natural antimicrobial agent in food safety.

## 1. Introduction

Globally, foodborne outbreaks, caused by pathogens, constitute a serious threat to public health and cause a heavy burden to economic in health care (Meng et al., 2021). People are easy to get sick by eating foods contaminated with common foodborne pathogens, and the rate of poisoning caused by eating foods contaminated by microorganism accounts has exceeded 50% among foodborne diseases (Park et al., 2017; Huang et al., 2021). In order to inhibit the growth of microorganisms, avoid food contamination and prolong the shelf life, synthetic additives and antimicrobial agents are widely applied to food processing (Patra and Baek, 2016). However, the application of chemical synthetic preservatives to control foodborne pathogens in the food industry may not be beneficial to microbial resistance and human health (Fleming-Jones and Smith, 2003; Calo et al., 2015; Schuh et al., 2020). Research has proven that artificial preservatives can cause serious health hazards such as hypersensitivity, allergy, asthma, hyperactivity, neurological damage and cancer (Kumari et al., 2019). For example, cytotoxic and genotoxic effects of potassium sorbate *via* inducing chromosome aberrations, sister chromatid exchange and DNA breakage (Dehghan et al., 2018). At present, thermal sterilization is widely used to ensure food safety. However, excessive heating can destroy heat-sensitive nutrients in foods (Mangundayao and Yasurin, 2017). Consequently, the development of high security and biological activities of natural antimicrobials is an excellent strategy for avoiding problems.

The extraction of bioactive compounds from plant resources has always attracted much attention in scientific research (Şeker Karatoprak et al., 2017; Gonelimali et al., 2018). As is known to all, flavonoids are a kind of polyphenols and belong to heterocyclic organic compounds containing more than 4,000 bioactive substances. And flavonoids are rich in tea, wine, honey, and nuts (Fathima and Rao, 2016). Moreover, several previous studies have suggested that plant flavonoids possess excellent biological properties including antibacterial, antioxidant, antiviral, anti-tumor, anti-aging, and other pharmacological actions (Ferreira et al., 2015; Pavlova et al., 2016; Zhang et al., 2021). Flavonoids could exert antibacterial activity *via* damaging the cytoplasmic membrane, inhibiting energy metabolism, and inhibiting the synthesis of nucleic acids, so flavonoids are considered constitutive antibacterial substances (Tan et al., 2022). *Chimonanthus salicifolius* S. Y. Hu. is a common herb extensively cultivated in Anhui, Hubei and Jiangxi provinces of China. Some studies have indicated that *C. salicifolius* S. Y. Hu. has many biological activities, such as anti-hyperglycemic, anti-inflammatory, anticancer and antimicrobial, with flavonoid belonging to one of the main active components (Chen et al., 2017; Sun, Q. et al., 2017). Particularly, previous studies have revealed the inhibition effect of ethanolic extracts of *C. salicifolius* S. Y. Hu. leaves on three bacteria and four fungi (Wang et al., 2018), and the antibacterial mechanism of extracts combined with streptomycin on two common foodborne pathogens (Wang, N. et al., 2020). However, up to date, the antibacterial activity and antibacterial mechanism of flavonoids from *C. salicifolius* S. Y. Hu. (FCS) alone have not been well studied. Furthermore, the antibacterial action modes and signaling pathways at the molecular level remain unclear.

In recent years, various molecular tools and bioinformatics techniques with accuracy and efficiency have been widely applied to identify and analyze the interaction between antibacterial agents and bacteria (Park et al., 2019). With genome sequencing and genomic technologies developing rapidly, transcriptome enables us to globally explore the complete responses at a transcriptional level (Chen et al., 2019). So far, transcription analysis has already been used to investigate the antibacterial mechanism of numerous bacteriostats, including natural plant ingredients, antibiotics and nanomaterials (Petek et al., 2010; Sun, D. et al., 2017; Liu et al., 2020; Kafantaris et al., 2021). The most effective way for transcriptomics is RNA sequencing (RNA-Seq), which is based on deep sequencing technologies and allows for more precise measurements of transcript levels and their isoforms than other methods (Wang et al., 2009; Hrdlickova et al., 2017).

Therefore, to provide more guidelines for the application of *C. salicifolius* S. Y. Hu. leaves and its flavonoid in the food industry, the present study aimed at determining the antibacterial effect of FCS on three Gram-negative bacteria including *Escherichia coli* (*E. coli*), *Salmonella enteritidis* (*S. enteritidis*), and *Pseudomonas aeruginosa* (*P. aeruginosa*) and three Gram-positive bacteria including *Staphylococcus. aureus* (*S. aureus*), *Bacillus subtilis* (*B. subtilis*), and *Listeria monocytogenes* (*L. monocytogenes*). Then the potential mechanism in charge of antibacterial activity against *S. aureus* was further detected. Additionally, the underlying action mode at the molecular level that involved inhibition effects of FCS on *S. aureus* was revealed for the first time by transcriptome analysis according to the differential gene expression.

## 2. Materials and methods

### 2.1. Chemicals and bacterial strains

Standards, including rutin, hyperin, isoquercitrin, luteoloside, kaempferol-3-*O*-rutinoside, luteolin-5-*O*-glucoside, astragalin, afzelin, quercetin, kaempferol, and the resazurin, solution were purchased from Shanghai Yuanye Bio-Technology Co., Ltd. (Shanghai, China). Acetonitrile (HPLC grade) was purchased from TEDIA Company Inc. (Shanghai, China) and ultra-Pure water (18.2 MΩ resistivity) was produced by a Milli-Q system (Millipore, Bedford, MA, United States). The alkaline phosphatase (AKP) kit and bicinchoninic acid (BCA) kit were purchased from Nanjing Jiancheng Bioengineering Institute (Nanjing, China). The bacterial genomic DNA extraction kit was purchased from Omega Bio-Tech (Norcross, United States). All other chemicals employed were of analytical grade. *Escherichia coli* (O157:H7 9490), *S. aureus* (ATCC 25923) and *S. enteritidis* (ATCC BAA-708) were provided by the Jiangxi Key Laboratory of Natural Products and Functional Food, Jiangxi Agricultural University. *Pseudomonas aeruginosa* (ATCC 9027), *L. monocytogenes* (ATCC 19115) and *B. subtilis* (GDMCC 1.784) were obtained from Guangdong Microbial Culture Center (GDMCC). Dimethyl sulfoxide (DMSO) was obtained from Beijing Solarbio Science & Technology Co., Ltd. (Beijing, China). Luria-Bertani broth (LB) was purchased from Qingdao Hope Bio-Technology Co., Ltd. (Qingdao, China). All tested strains were cultured on LB broth at 37°C.

### 2.2. Preparation of bacterial suspension

Before the experiments was carried out, all strains were stored in LB broth with 20% glycerol (v/v) at −80°C. The selected cultures were streaked on LB agar and incubated at 37°C for 24 h. Then a single colony of each strain was inoculated into LB broth, followed by incubation at 37°C for 12 h for subsequent study. After that, bacteria were diluted 3:100 (v/v) into fresh LB broth and cultured to the logarithmic phase. The cells were enriched through centrifugation, then washed and mixed with 0.1 M sterile Phosphate buffer saline (PBS, pH 7.4) to achieve the concentration which was monitored by the absorbance value at 600 nm and the plate count method.

### 2.3. Preparation of FCS

The dried leaves of *C. salicifolius* S. Y. Hu. were bought from a farmer in Lishui City, Zhejiang Province, China. The identity of the plant sample was identified and confirmed by Prof. Zhiyong Zhang at the College of Agricultural Science, Jiangxi Agricultural University. The powder of the sample should be less than 60 mesh which was pulverized by a mixer mill. In short, referring to the previously reported method (Chen et al., 2017), the powder was extracted twice in 60% ethanol with a ratio of 1:20 (m/v) and homogenized by ultrasonication at 55°C. Then the liquid was concentrated in a vacuum and purified on D1400 macroporous resin by washing it with distilled water and eluting it with 40% ethanol. Finally, the ethanol extracts (FCS) were obtained after being concentrated and lyophilized. The calculation of total flavonoids content was performed by the method prescribed by Lei et al. (2019).

### 2.4. Chemical composition of FCS

To figure out the chemical composition of FCS, the procedures were performed through a C_18_ column (250 mm × 4.6 mm, 5 μm, Waters, Ireland) based on high-performance liquid chromatography (HPLC; Chen et al., 2017). The sample of FCS and flavonoid standards were obtained by the 0.22 μm filter membrane after dissolution into methanol. The mobile phases were 0.1% acetic acid-water (A) and acetonitrile (B). Gradient elution was performed as followed: 0–15 min, from 90 to 75% B; 15–30 min, from 75 to 60% B; 30–40 min, from 60 to 50% B. Moreover, the injection volume: 10 μl; the flow rate: 1.0 ml/min. The LOD and LOQ were obtained from calibration curves according to the formulations: LOD = 3.3 *σ*, LOQ = 10 *σ*, where *σ* was defined as the ratio of the standard deviation of the response to the slope of the calibration curve, respectively (Jovanović et al., 2021).

### 2.5. Antibacterial activity assays

#### 2.5.1. Inhibition zone assay

The antimicrobial effect of FCS on the six bacteria was investigated by the oxford cup method (Kang et al., 2018). The sample of FCS was prepared by dissolving in 2% DMSO to reach a certain concentration of 100 mg/ml and sterilizing through a 0.22 μm microporous organic membrane. After that, the LB agar plates were uniformly covered by 0.1 ml prepared bacterial suspension, and then put the oxford plates (6 mm in diameter) on plates at equal distances. Subsequently, 0.1 ml of prepared FCS solution was added to the oxford plates. The inhibition zones against the selected strains were observed after a period of incubation of 24 h at 37°C.

#### 2.5.2. MIC and MBC assay

The reference method to determine MIC and MBC was reported by Wang et al. (2018). In brief, FCS was prepared and serially diluted in LB by two-fold dilution method in a sterile 96-well plate, then 0.1 ml approximately 10^6^ CFU/ml of bacterial suspensions was added to obtain concentrations of 0.78, 1.56, 3.12, 6.25, 12.5, 25, and 50 μg/μl. After the mixture was incubated at 37°C for 18 h, to each tube of the microplate, an appropriate volume of resazurin solution (0.05% w/v) was added, then incubated at 37°C for 1 h sequentially. Holes were measured visually: a color change from blue to pink or mauve, was taken as representative of bacterial reproduction; while the highest dilution which remained blue was used to represent the MIC in contrast. The MIC was defined as the minimum concentration of FCS that demonstrated no obvious color change. The MBC was determined by taking 100 μl of mixtures from uncolored holes to spread on LB agar plates and incubating at 37°C for 24 h. The minimum concentration at which showed no obvious bacteria growth was defined as the MBC. *Staphylococcus aureus*, which is belong to Gram-positive bacteria and have the most sensitive effect to FCS among tested strains, was selected for the following experiments.

### 2.6. Antibacterial mechanism assays

#### 2.6.1. Effects on the bacterial growth curve

Firstly, the antibacterial mechanism of FCS was evaluated by the bacterial growth curve (Wang, N. et al., 2020). The preparations of bacterial suspensions and samples were based on Section 2.2 and Section 2.3, respectively. Then, the cells of *S. aureus* with FCS at MIC were cultured at 37°C on a rotary shaker at the speed of 180 r/min. Control groups were prepared with 2% DMSO which is the solvent of FCS. At regular intervals, the absorbance values at 600 nm from 0 to 10 h were monitored to determine the effect of FCS on bacterial growth.

#### 2.6.2. Effects on the bacterial cell wall

The antibacterial mechanism of FCS on the bacterial cell wall integrity of *S. aureus* was determined as previously described (Xu, M. et al., 2019). The prepared bacterial suspension and sample of FCS as shown in Section 2.2 and Section 2.3, respectively. In brief, the cells of *S. aureus* at the logarithmic phase in LB broth were enriched through centrifugation at 6,000 *g* for 10 min, washed and resuspended in PBS to 10^6^ CFU/ml. Then, the prepared bacterial suspension with FCS treatment at MIC was cultured with shaking at 37°C for 6 h. With the method of centrifugation separation, the supernatant was obtained and the AKP content was measured based on the manufacturer’s instructions.

#### 2.6.3. Effects on the cytoplasmic membrane

The cytoplasmic membrane permeability was investigated by detecting the loss of intracellular components including proteins and nucleic acids (Wang J. et al., 2020). The incubation of *S. aureus* at 37°C with FCS (MIC) lasted for 6 h. At 0, 0.5, 1.0, 2.0, 3.0, 4.0, 5.0, and 6.0 h, the mixture with the right amount was taken and centrifuged at 6,000 *g* for 10 min, then the supernatants were obtained after filtering by 0.22 μm membrane. The method to detect the release of protein was modified from the description by Cui et al. (2018a), and the leakage of nucleic acids was determined with the OD_260_ of each supernatant (Zhu and Zhang, 2020).

#### 2.6.4. DNA damage

The antibacterial action of FCS on the genomic DNA of *S. aureus* was determined by agarose gel electrophoresis (AGE; Yang et al., 2020). Prepared freshly bacterial suspension of *S. aureus* was cultured with different concentrations of FCS at 37°C for 8 h. Only 2% DMSO was determined as a blank control group. The extraction of bacterial genomic DNA of *S. aureus* was performed by a bacterial genomic DNA extraction kit following the manufacturer’s instructions. Finally, the extracted genomic DNA was separated by AGE (1.0%, w/v) after the purity of genomic DNA samples was ascertained.

#### 2.6.5. Transmission electron microscope observation

The changes in microscopic morphologies caused by FCS against *S. aureus* were measured based on previous reports (Fei et al., 2018; Kang, J. et al., 2020). The bacterial cells with different treatments were cultured at 37°C for 6 h and then separated by centrifugation. The precipitated cells were washed in PBS three times and mixed with 2.5% (v/v) glutaraldehyde at 4°C overnight. Furthermore, the fixed cells were washed repeatedly using phosphate buffer saline (PBS) and dehydrated with ethanol of gradient concentration (30, 50, 70, 90, and 100%), and finally used anhydrous acetone to replace the ethanol. After a series of processing including embedding and cutting into slices (70 nm), the microscopic morphologies of cells were viewed using transmission electron microscopy (TEM; H-7650, Hitachi, Tokyo, Japan).

#### 2.6.6. RNA extraction, library construction, and sequencing performance

To provide further insight into the transcriptional changes of bacteria, the freshly prepared bacterial suspension of *S. aureus* was exposed to a low concentration of FCS (1/2 MIC) at 37°C for 3 h, meanwhile, taken the sample solvent as a negative control. The bacterial cells were centrifuged at 6,000 *g* for 10 min and washed three times with sterile PBS. Subsequently, the cells were enriched by centrifugation at 14,000 *g* for 1 min, frozen by liquid nitrogen for 4 h, and kept at −80°C until required. The total RNA of bacteria was extracted by the mirVana miRNA Isolation Kit (Ambion), and its integrity and quality were detected through AGE and Bioanalyzer 2,100 (Agilent Technologies, CA, United States). The samples with RNA Integrity Number (RIN) ≥ 7 were subjected to the subsequent analysis. The libraries were constructed using TruSeq Stranded Total RNA with Ribo-Zero Gold according to the manufacturer’s instructions. Illumina sequencer was used for sequencing after the constructed library was qualified by Agilent 2100 Bioanalyzer. RNA-Seq analysis was completed by Shanghai OE Biomedical Science and Technology Company (Shanghai, China).

#### 2.6.7. RNA-Seq data analysis

In transcriptome sequencing analysis, the expression level of genes was estimated by locating the count of sequencing sequence (reads) in the genome area or the exon region of the gene. Clean data (clean reads) were obtained by removing adapter-containing, poly-N, and low-quality reads from the raw data (raw reads) using in-house Perl scripts. All downstream analyses were based on clean data with high-quality. Then, using Rockhooper2 (Tjaden, 2015) to align clean reads to the reference genome of the experimental specie, the sample should be assessed by genomic and gene alignment. Furthermore, using the estimate Size Factors function of the R package of DESeq to standardize counts, and use the nbinomTest function to calculate the *p*-value and foldchange of difference comparison. Pick out the difference transcripts that *p*-value less than 0.05 and Difference of multiples more than 2, and the GO and KEGG enrichment analysis of the differential genes was performed by hypergeometric distribution tests to determine the biological functions or pathways that are mainly affected by differential genes.

#### 2.6.8. RT-qPCR verification

The accuracy of RNA-Seq analysis was validated by RT-qPCR assay, which was completed by the procedure used in transcriptome analysis. Seven selected DEGs were used to confirm the RNA-Seq dates and the relative fold changes were calculated after normalization using the comparative CT method. The thermal cycling parameters were as follows: initial denaturation at 94°C for 30 s, followed by 45 cycles of 94°C for 5 s and 60°C for 30 s. Each sample was run in triplicate for analysis. Meanwhile, the expression level of 16S rRNA was defined as the internal control.

### 2.7. Statistical analysis

All assays including the exploration of antibacterial activity and relatively potential mechanism were repeated in triplicate and the data of results was present in mean value with standard deviation (SD). All significant differences (*p* < 0.05, 0.01, and 0.001, respectively) were analyzed using one-way analysis of variance (ANOVA) and LSD test by SPSS software (version 25.0; IBM Corp., Armonk, NY, United States).

## 3. Results

### 3.1. Identification of FCS

**Figure 1:**
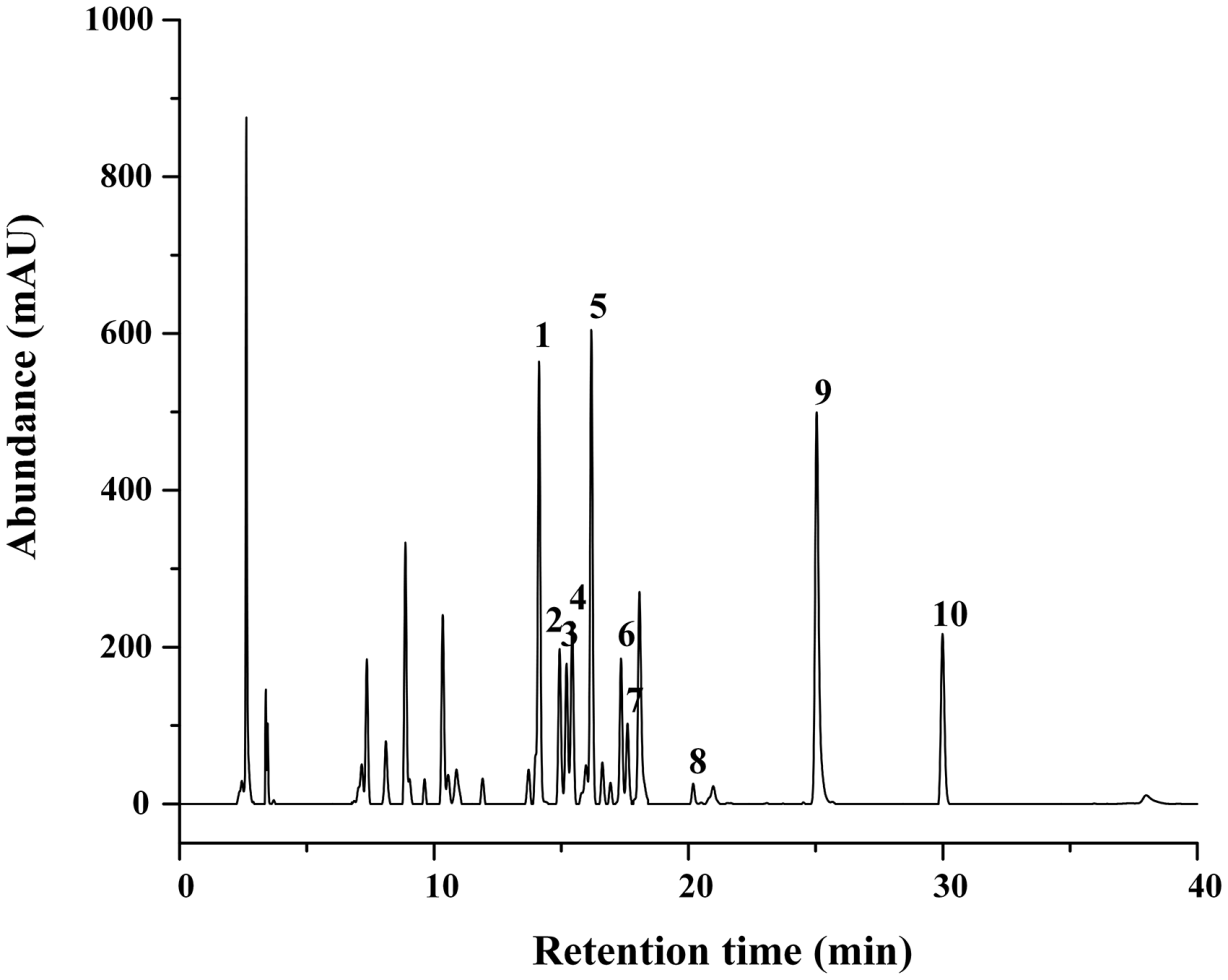
HPLC Chromatograms of FCS. (1) Rutin; (2) hyperin; (3) isoquercitrin; (4) luteoloside; (5) kaempferol-3-*O*-rutinoside; (6) luteolin-5-*O*-glucoside; (7) astragalin; (8) afzelin; (9) quercetin; (10) kaempferol.

The aluminum chloride colorimetric method estimated that the flavonoid content of FCS from *C. salicifolius* S. Y. Hu. was 84.2 ± 2.0%, which was determined by the rutin standard equation: *Y* = 0.8161*X* + 0.0259, *R*^2^ = 0.9967, where *Y* represents the absorbance value and *X* represents the flavonoids content in mg/ml. Then, the flavonoid compound characterization of FCS was accomplished by HPLC. From Table 1, the component from FCS with the highest content was kaempferol-3-*O*-rutinoside (142.18 ± 2.36 μg/ml), followed by kaempferol (123.79 ± 0.29 μg/ml) and rutin (122.34 ± 5.49 μg/ml). As shown in Figure 1, Peak 1 (tR = 14.10 min, rutin); Peak 2 (tR = 14.90 min, hyperin); Peak 3 (tR = 15.17 min, isoquercitrin); Peak 4 (tR = 15.40 min, luteoloside); Peak 5 (tR = 16.16 min, kaempferol-3-*O*-rutinoside); Peak 6 (tR = 17.33 min, luteolin-5-*O*-glucoside); Peak 7 (tR = 17.59 min, astragalin); Peak 8 (tR = 20.18 min, afzelin); Peak 9 (tR = 25.03 min, quercetin); and Peak 10 (tR = 29.95 min, kaempferol).

**Table 1:** Chemical composition of flavonoids from *Chimonanthus salicifolius* S. Y. Hu.

Peak	Compounds	Calibration curve	*R* ^2^	FCS (μg/ml)	LOD	LOQ
1	Rutin	y = 34.283x + 54.442	0.9997	122.34 ± 5.49	0.485	1.600
2	Hyperin	y = 27.021x + 91.082	0.9999	47.52 ± 0.36	0.041	0.134
3	Isoquercitrin	y = 24.663x + 121.2	0.9999	41.35 ± 1.26	0.154	0.509
4	Luteoloside	y = 24.161x + 220.83	0.9992	61.12 ± 1.2	0.015	0.050
5	Kaempferol-3-*O*-rutinoside	y = 28.814x + 93.511	0.9992	142.18 ± 2.36	0.248	0.819
6	Luteolin-5-*O*-glucoside	y = 17.629x + 11.631	0.9995	78.32 ± 0.17	0.030	0.099
7	Astragalin	y = 19.06x + 22.263	0.9999	42.75 ± 1.85	0.294	0.970
8	Afzelin	y = 15.343x − 135.76	0.9954	22.90 ± 0.13	0.027	0.088
9	Quercetin	y = 68.872x − 47.16	0.9990	78.46 ± 0.22	0.009	0.031
10	Kaempferol	y = 18.582x + 41.227	0.9997	123.79 ± 0.29	0.047	0.154

Results are expressed as mean ± SD of three determinations. LOD, limit of detection; LOQ, limit of quantification.

### 3.2. Antibacterial activity

At first, the oxford cup method was used to determine the antibacterial activity of FCS. As shown in Table 2, FCS could effectively inhibit the tested bacteria including *S. aureus*, *B. subtilis*, *L. monocytogenes, E. coli,* and *S. enteritidis*, with the values of DIZ were 15.93 ± 2.63 mm, 12.46 ± 0.76 mm, 11.89 ± 1.16 mm, 12.21 ± 1.06 mm, and 11.35 ± 0.23 mm, respectively. However, there were no inhibition effects on *P. aeruginosa* (Gram-negative bacteria) in the tested concentration. The reason for this situation may lie in that *P. aeruginosa* is not sensitive to FCS with a concentration of 100 mg/ml. Then, the broth microdilution method was performed to determine the values of MIC and MBC of FCS. As shown in Table 2, the MICs of FCS against *S. aureus*, *B. subtilis*, *L. monocytogenes, E. coli, S. enteritidis*, and *P. aeruginosa* were 1.56, 6.25, 6.25, 6.25, 12.5, and 25 mg/ml, respectively. In addition, the MBCs of FCS against *S. aureus*, *B. subtilis*, and *L. monocytogenes* were 6.25, 50, and 50 mg/ml, the MBCs of FCS against *E. coli, S. enteritidis*, and *P. aeruginosa* were higher than 50 mg/ml, respectively. These results illustrated that FCS has potential antibacterial activity and has a more significant effect on gram-positive bacteria than on gram-negative bacteria.

**Table 2:** Antibacterial activities of flavonoids from *Chimonanthus salicifolius* S. Y. Hu. against six bacteria.

Microorganisms	Strain	FCS		
		DIZ (mm)	MIC (mg/ml)	MBC (mg/ml)
Gram-positive				
*Staphylococcus aureus*	ATCC 25923	15.93 ± 2.63	1.56	6.25
*Bacillus subtilis*	GDMCC 1.784	12.46 ± 0.76	6.25	50
*Listeria monocytogenes*	ATCC 19115	11.89 ± 1.16	6.25	50
Gram-negative				
*Escherichia coli*	O157:H7 9,490	12.21 ± 1.06	6.25	>50
*Salmonella enteritidis*	ATCC BAA-708	11.35 ± 0.23	12.5	>50
*Pseudomonas aeruginosa*	ATCC 9027	–	25	>50

“–” means no inhibition zone.

### 3.3. Antibacterial mechanism

#### 3.3.1. Bacterial growth curve analysis

**Figure 2:**
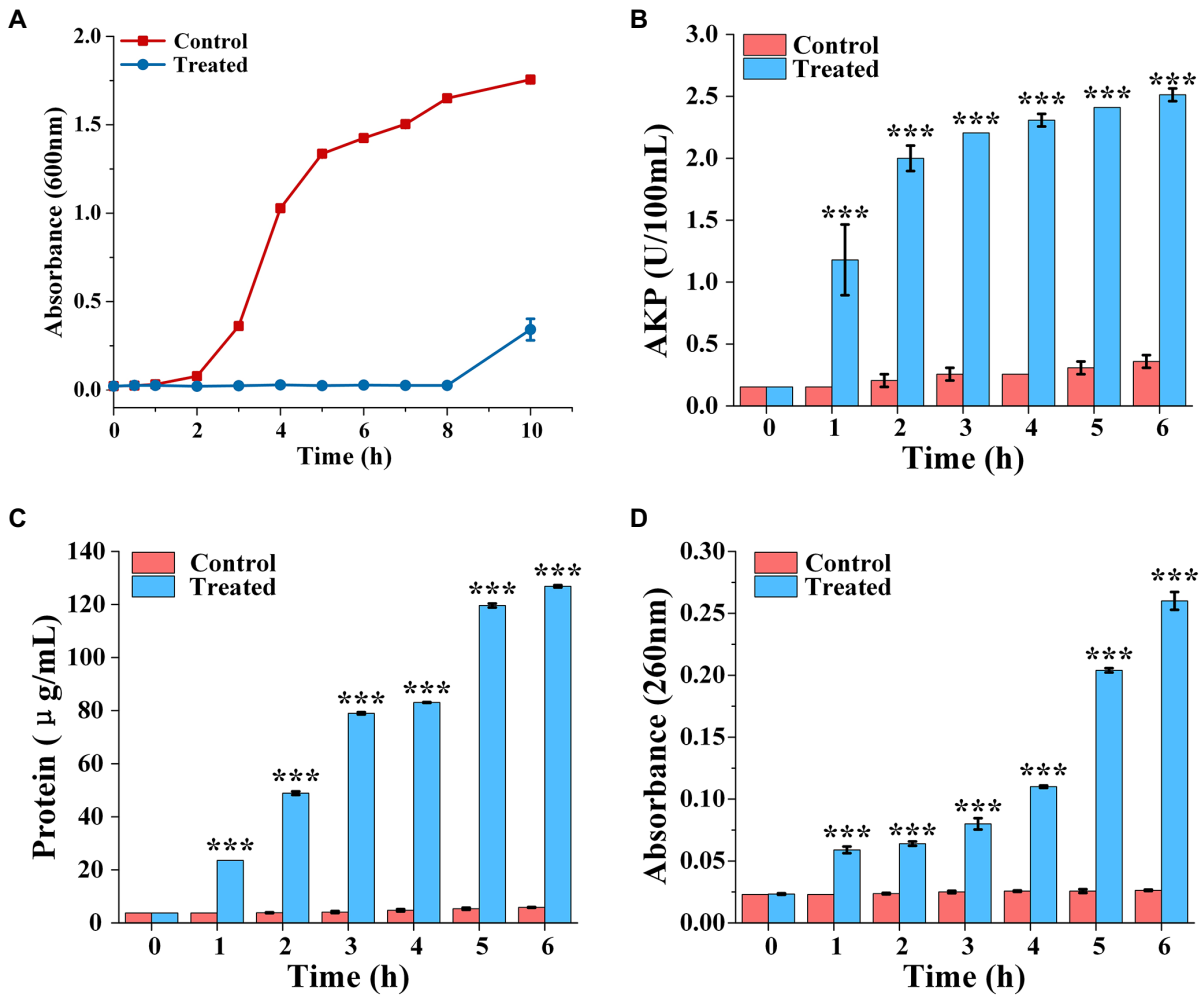
The growth curve of *Staphylococcus aureus* treated with FCS **(A)**. Determination of extracellular AKP content of *S. aureus* after FCS treatment **(B)**. The release of protein from *S. aureus* cells after FCS treatment **(C)**. The leakage of nucleic acids from *S. aureus* after FCS treatment **(D)**. *** (three asterisks) represents highly significant differences (*p* < 0.001) in the comparison between the Control and the Treated.

The bacterial growth curves in the presence of FCS were plotted to determine the rhythm of growth and propagation under specific conditions. As observed in Figure 2A, cells of the control group grew slowly during the beginning 2 h, then stepped into the stabilization phase after through rapid growth logarithmic phase and adjusted phase. However, the final bacterial concentration of *S. aureus* treated with FCS (MIC) after 10 h was decreased to 5/16 compared to the control. The results were consistent with those of Lin et al. (2020), who proved that flavonoids from *Ageratum conyzoides* L. have inhibition effects on the growth of *E. coli*, *S. aureus*, and *P. aeruginosa*. Taken together, the results suggested that inhibition of bacterial growth is one of the potential antibacterial actions of FCS on *S. aureus*.

#### 3.3.2. Effects on the cell wall

The release of AKP from bacteria was detected to monitor whether the bacterial cell wall is damaged. As shown in Figure 2B, the extracellular AKP activity of control group had no obviously change. However, the extracellular AKP activity with FCS treatment increased at the first hour and was significantly higher than the blank control (*p <* 0.05). After 6 h, extracellular AKP content of *S. aureus* with FCS treatment were increased to 6.97 folds compared with the control group. The results suggested that FCS damaged the integrity of the cell wall.

#### 3.3.3. Effects on the cell membrane

The effects of FCS on cell membrane of *S. aureus* are represented in Figure 2C, which shown that a persistent and distinct escape of protein from samples in FCS treatment within 6 h, following the protein concentration on *S. aureus* increased from 5.85 ± 0.14 to 126.86 ± 0.43 μg/ml. On the contrary, the protein concentration only has a slight change in control groups compared to cells with FCS treatment. As shown in Figure 2D, the content of the nucleic acids in the extracellular environment had significant differences compared to the control over time (*p <* 0.05). FCS lead to an 8.87-fold increase in the absorbance value of cells in 260 nm compared to blank control after cultured continuously for 6 h. The above results demonstrated that FCS could destroy the cytoplasmic membrane permeability and cause the loss of intracellular substance, which may make a bad effect on maintaining the normal physiological functions of bacteria and even lead to cell death.

#### 3.3.4. Effects on the bacterial genomic DNA

**Figure 3:**
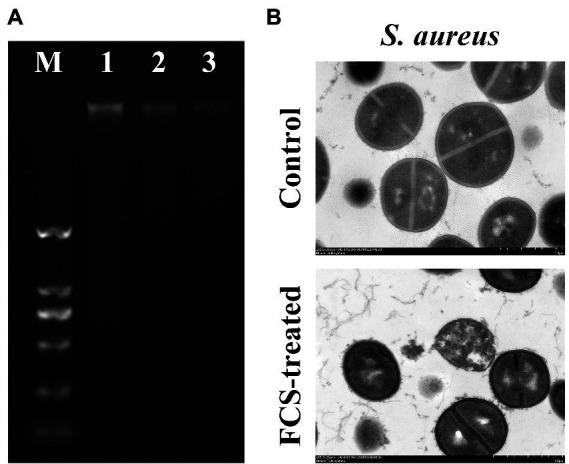
The effects of FCS on Genomic DNA of *Staphylococcus aureus* **(A)**. Lane M: DL2000 DNA marker; Lane 1: the control group of *S. aureus*; Lane 2–3: *S. aureus* with FCS treatment at MIC and MBC, respectively. The TEM images of *S. aureus* **(B)**.

To evaluate the impacts of FCS on the genomic DNA of *S. aureus*, the AGE was performed in the present study. As shown in Figure 3A, the clear and bright DNA bands (Lane 1) of the genomic DNA electrophoretogram could be visualized for cells without FCS treatment (i.e., blank control). However, in the treated group with different concentrations of FCS, DNA bands (Lane 2 and 3) faded or even lost, illustrating that FCS could destroy the DNA structures of *S. aureus*, with this destructive effect in a concentration-dependent manner of FCS.

#### 3.3.5. TEM observation

To further confirm the antibacterial action mode of FCS on the intracellular organization of cells visually, the control and FCS-treated samples were examined by TEM. According to Figure 3B, the image of control samples appeared normal and clear with intact cell membranes and uniformly distributed cytoplasm. However, the bacterial ultrastructure of *S. aureus* showed an irregular shape, blurry membranes and the leakage of cytoplasmic after 6 h of FCS treatment. The results of TEM observation were consistent with the previous results in this study, which further suggested that FCS could destroy cell wall and membrane, cause leakage of cell contents and even lead to cell death.

### 3.4. RNA sequencing analysis

#### 3.4.1. Sequence analysis

All the above results have suggested that the inhibition effects against *S. aureus* of FCS were mainly achieved by destroying the bacterial cell wall and cytoplasmic membrane and causing DNA damage. Thus, we assessed the specific response at the mRNA level by transcriptome analysis to further explore the antibacterial mechanism of FCS against *S. aureus*. After filtering through the raw reads, there were 14.15 G clean dates and the Q30 values were >95% (Q30 represents 1,000 of the sequencing error rate), suggesting that the data were of high quality. Detailed data are shown in Supplementary Table S1.

#### 3.4.2. Identification of DEGs

**Figure 4:**
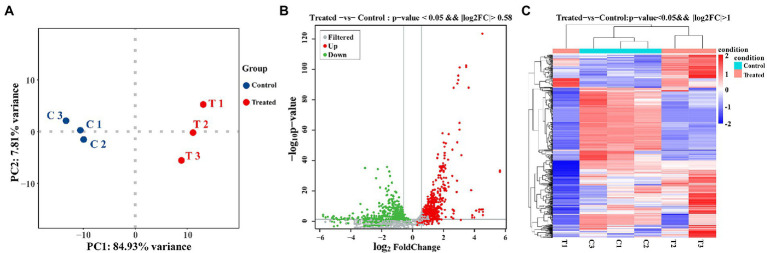
PCA analysis between the group with FCS treatment and the control group **(A)**. The volcano diagram of DEGs after FCS treatment compare to the control **(B)**. Cluster analysis of DEGs after FCS treatment, in which red cluster represents up-regulated and blue cluster represents down-regulated **(C)**.

In this study, transcriptome combined with bioinformatics analysis was conducted for the first time to figure out the antibacterial action mode of FCS on *S. aureus*. The differentially expressed genes (DEGs) in response to the treatment with FCS at 1/2 MIC (T1, T2, and T3) and the blank control (C1, C2, and C3) from *S. aureus* were identified. The criteria of significant differential expression were| log_2_ (fold change) | > 1 and False Discovery Rate (FDR) < 0.05. As illustrated in Figure 4A, the transcription levels of three repeat samples in the same group were slightly different, but significant differences were observed between the two groups, this result is well in agreement with that of previous antimicrobial experiments, determining the potential antibacterial effect of FCS. Furthermore, as we can observe in Figure 4B, 671 DEGs were identified in samples after FCS treated (|log_2_ (fold change)| > 1, FDR < 0.05), with 338 and 333 genes showing up-regulation and down-regulation, respectively, compared to the control. The most significantly upregulated gene was transcript-38,652 (log_2_FC = 5.60). Besides, the expression of 10 genes was downregulated with more than five-fold changes. The top two downregulated genes were SAOUHSC_00913 (log_2_FC = −5.84) and SAOUHSC_00396 (log_2_FC = −5.60), which were related to DNA binding and hypothetical protein, respectively. A complete description of DEGs belonging to *S. aureus* between the FCS treatment and the control groups is shown in Supplementary Table S2. Additionally, as observed in Figure 4C, the metabolic pathways of cells with different treatments were inconformity. These results suggest the relatively strong antibacterial effect of FCS on *S. aureus*. Consequently, the expression of the differential gene in *S. aureus* will be studied to explore the antibacterial action mode of FCS at the molecular level.

#### 3.4.3. GO enrichment analysis

**Figure 5:**
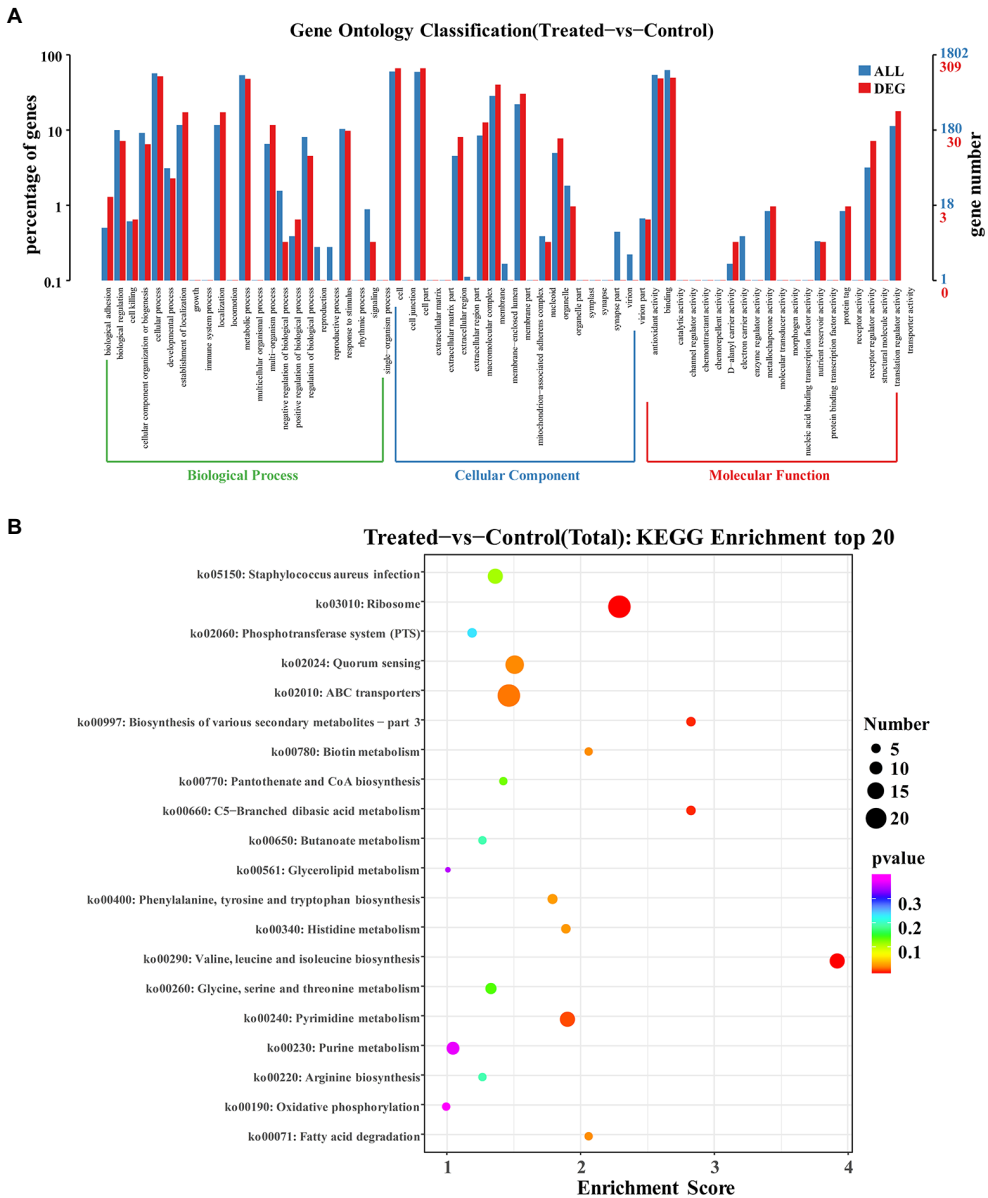
GO enrichment analysis of DEGs in *Staphylococcus aureus* after the action of 1/2 MIC FCS **(A)**. KEGG enrichment analysis of DEGs in *S. aureus* after the action of 1/2 MIC FCS **(B)**.

Gene Ontology (GO) analysis is considered an internationally standardized system for classifying gene function, and it provides strictly defined concepts and controlled vocabulary to reveal the gene properties and gene products in any organism (Xiu et al., 2015). GO term enrichment analysis was performed on the differential genes of the group with FCS treatment and the control group. As Figure 5A illustrated, GO enrichment analysis shows a total of 23 terms related to biological processes (BP), 20 terms for cellular components (CC), and 21 terms for molecular functions (MF), respectively. Regarding BP ontology, the most represented categories were cellular process (GO:0009987) and metabolic process (GO:0008152). In the ontology of CC, cell (GO:0005623), cell part (GO:0044464), and membrane (GO:0016020) were significantly enriched. Within MF ontology, the main groups that DEGs distributed were catalytic activity (GO:0003824), binding (GO:0005488), and transporter activity (GO:0005215). The results showed that many genes involved in cellular components were altered, suggesting that exposure to FCS caused changes in cell structure and cellular components. In addition, FCS affected the metabolism and activity of *S. aureus*.

#### 3.4.4. KEGG pathway analysis

The research of biological pathways is important for understanding and promoting genomics research. The highly integrated database Kyoto Encyclopedia of Genes and Genome (KEGG) provides data on biological systems and their relationships at the molecular, cellular, and organism levels (Sun, D. et al., 2017). Based on bioinformatics databases, DEGs were categorized into different pathways by KEGG pathways significant enrichment analysis (Figure 5B). KEGG analysis revealed 20 KEGG pathways, among which, ABC transporters (ko02010) and ribosome (ko03010) pathways were the most enriched pathways in the DEGs, and other major pathways included quorum sensing (ko02024), *Staphylococcus aureus* infection (ko05150), valine, leucine and isoleucine biosynthesis (ko00290), pyrimidine metabolism (ko00240), and purine metabolism (ko00230), indicating that these pathways played significant roles in antibacterial processes of FCS.

### 3.5. Validation of RNA-Seq expression by RT-qPCR

**Figure 6:**
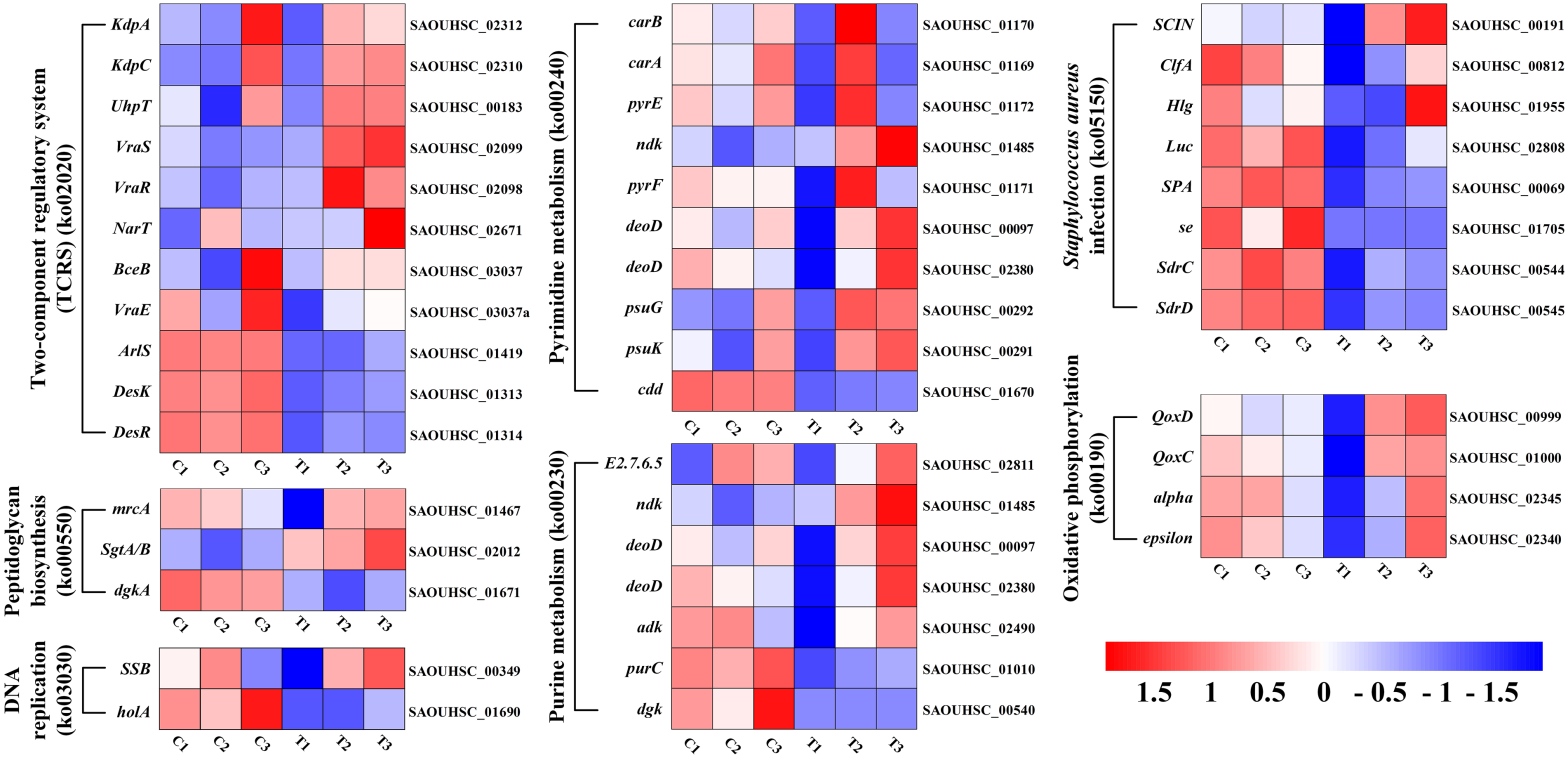
Heat map representation of gene expression after FCS treatment relative to two-component regulatory system, peptidoglycan biosynthesis, DNA replication, pyrimidine metabolism, purine metabolism, *Staphylococcus aureus* infection and oxidative phosphorylation.

The DEGs expression levels of the same RNA samples were detected by RT-qPCR to verify the RNA-Seq accuracy. As we can observe in Figure 6, the expression levels of SAOUHSC_03006, SAOUHSC_01879, transcript-32294, and transcript-32305 were downregulated, while the expression levels of transcript-38644, transcript-38651, and transcript-39137 were upregulated. Therefore, the results of the RT-qPCR assay were consisted with the transcriptome analysis, illustrating that the RNA-Seq data was valid.

## 4. Discussion

Understanding FCS’s antibacterial activity and action mode will aid in its use in the food industry and boost the added value of the leaves of *C. salicifolius* S. Y. Hu., which is critical for regional economic development. In the present study, the flavonoids from FCS were prepared by ultrasound-assisted alcohol extraction, purified by microporous resins. Then the flavonoid content was measured by ultraviolet spectrometry and the composition was determined by HPLC. The antibacterial activity of FCS against three Gram-positive bacteria and three Gram-negative bacteria was evaluated by DIZ, MIC, and MBC. As Table 1 shown, the tendency of MICs and MBCs in accord with the result of DIZ, which was proved that FCS had a more significant effect on gram-positive bacteria than gram-negative bacteria. The main reason for that may be as follows: the gram-positive bacteria only has a single-layer membrane structure, while the gram-negative bacteria have a double-layer membrane structure, which can hedge the penetration of antibacterial substances and stimulate the immune function of cells (Valkenburg et al., 1985). Flavonoids also showed antibacterial activity on a wide range of gram-negative and gram-positive bacteria, and fungi (Simard et al., 2016; Xu, Z. et al., 2019). For example, the flavonoids in chestnut had antibacterial effects on common spoilage bacteria in low-temperature meat products such as *Proteus* and *Macrococcus* (Zhang, W. et al., 2020). Flavonoids in etiolated tissues from fresh-cut Chinese water-chestnuts (*Eleocharis tuberosa*) had been reported to be effective against *B. subtilis*, *S. aureus*, *E. coli*, and *Salmonella* (Yuan et al., 2022). Other research has reported that the antibacterial effect and mode of action of flavonoids from Licorice against methicillin-resistant *S. aureus* (Wu et al., 2019). Additionally, *S. aureus* was the most sensitive bacteria among the tested strains to FCS, with DIZ was 15.93 ± 2.63 mm, MIC was 1.56 mg/ml and MBC was 6.25 mg/ml. Therefore, we will explore the potential mechanism to respond to the inhibitory effects of FCS on *S. aureus*, which is strongly pathogenic in the food industry.

Bacterial cell walls are hard structures surrounding the cells, which can maintain the cell morphology, withstand pressure and reduce the damage caused by foreign materials (Wang, F. et al., 2017). AKP is related to many microbial biosynthesis and metabolic processes, such as phosphate transfer and metabolism, protein secretion and synthesis, and calcium metabolism (Lansdown, 2010; Wang et al., 2015). In general, AKP is unable to pass through intact bacterial cell walls because it is present between the bacterial cell wall and cell membrane (Pramanik et al., 2017). However, AKP concentration in the extracellular environment will increase if the cell wall integrity is destroyed (Diao et al., 2018). After 6 h, the extracellular AKP content of *S. aureus* with FCS treatment were increased to 6.97 folds compared with the control group. This result was consistent with those of Kang, S. et al. (2020), who proved that the extracellular AKP activity of *Pseudomonas fluorescens* and *Methicillin-resistant Staphylococcus aureus* treated with lactobionic acid rapidly increased within 2 h, indicating that the bacterial cell wall was destroyed rapidly. Therefore, we speculated that the antibacterial action mode of FCS may link with the destruction of the cell wall.

The cell cytoplasmic membrane performs a crucial role in avoiding the influence of the external environment and carrying nutrients to support bacterial normal life (Liu et al., 2018). Some papers reviewed that small molecules and several macromolecules such as proteins and nucleic acids would be let out if the cytoplasmic membrane was damaged (Wang, F. et al., 2017; Sun et al., 2018). The absorption at 260 nm is detected to measure the DNA and RNA contents whose absorption maximum is at 260 nm (Liu et al., 2016; Zhao et al., 2021). Therefore, the analysis of the possible impact of FCS on bacterial cell membrane permeability was detected by the protein concentration and 260 nm-absorbing material of the bacterial culture. The protein content and absorbance value at 260 nm of groups with FCS treatment were significantly higher than those in the control group (*p <* 0.05), and the maximum multiples of protein content and absorbance value at 260 nm were 20.69-fold and 8.87-fold, respectively, indicating that FCS could damage the permeability and integrity of the cell membrane. Similarly, Zhao et al. (2020) reported that the content of nucleic acid and protein in bacterial suspension increased after being treated with tea saponin, suggesting that tea saponin destroyed the integrity of the membrane and affected normal growth. Moreover, lots of natural antimicrobials that inhibit bacterial growth are controlled by the mode of membrane damage (Cui et al., 2018a, 2019; Zhang, L.-L et al., 2020), such as changing the cytoplasmic membrane permeability and integrity.

DNA is one of the most important genetic materials. The destruction of DNA may hinder gene expression, resulting in blocking the synthesis of normal enzymes and receptors and leading to cell death. The results of agarose gel nucleic acid electrophoresis showed that the DNA bands treated with FCS decreased or even disappeared, indicating that the expression of FCS-damaged bacterial genomic DNA decreased. Studies have reported that natural products damage DNA may by damaging the cell membrane structure, which leads to the leakage of nucleic acid and other macromolecules to the extracellular environment, or through forming a chimera with the bacterial DNA to destroy the double helix structure (Cui et al., 2018b). In addition, Sperotto et al. (2013) reported that natural products could reduce the activity of the DNA gyrase and ribonucleic acid synthetase, which is unfavorable to the synthesis and restoration of nucleic acid substances in cell. Furthermore, some reports have verified that the secondary structure and morphology of DNA may change by binding to the minor groove of genomic DNA with some bacteriostatic (Tao et al., 2016; Wang, L.-H. et al., 2017). Therefore, the above results proved that FCS could also damage the genomic DNA of sensitive strains except destroy the cell wall and membrane.

Previous researches have indicated that one of the most important factors that natural products effectively inhibit foodborne pathogens is that they can destroy the cellular morphology and make the intracellular component leak (Joshi et al., 2014; Fei et al., 2018). This analysis of TEM suggested that FCS has destructive actions on the bacterial cell wall and cytoplasmic membrane, then cause the leakage of cytoplasmic and even cell death. The results have coincided with the results reported by Wang, J. et al. (2020), who proved the same effects on *S. putrefaciens* cells treated by flavonoids from *Sedum aizoon* L. Chang et al. (2021) also revealed the effect of crude extract of chrysanthemum bud on the cell morphology of Cronolia.

With the development of research technology, the study of bacterial transcriptome plays an important role in revealing the pathogenic mechanism, the drug-resistance mechanism and the antibacterial mechanism of antibacterial agents. Transcriptome analysis reveals the action targets of antibacterial agents by analyzing the changes and differences of RNA in the sample. Therefore, RNA-Seq was used to analyze the changes in gene expression of *S. aureus* with 1/2 MIC FCS treatment at the mRNA level from the point of view of molecular biology.

**Figure 7:**
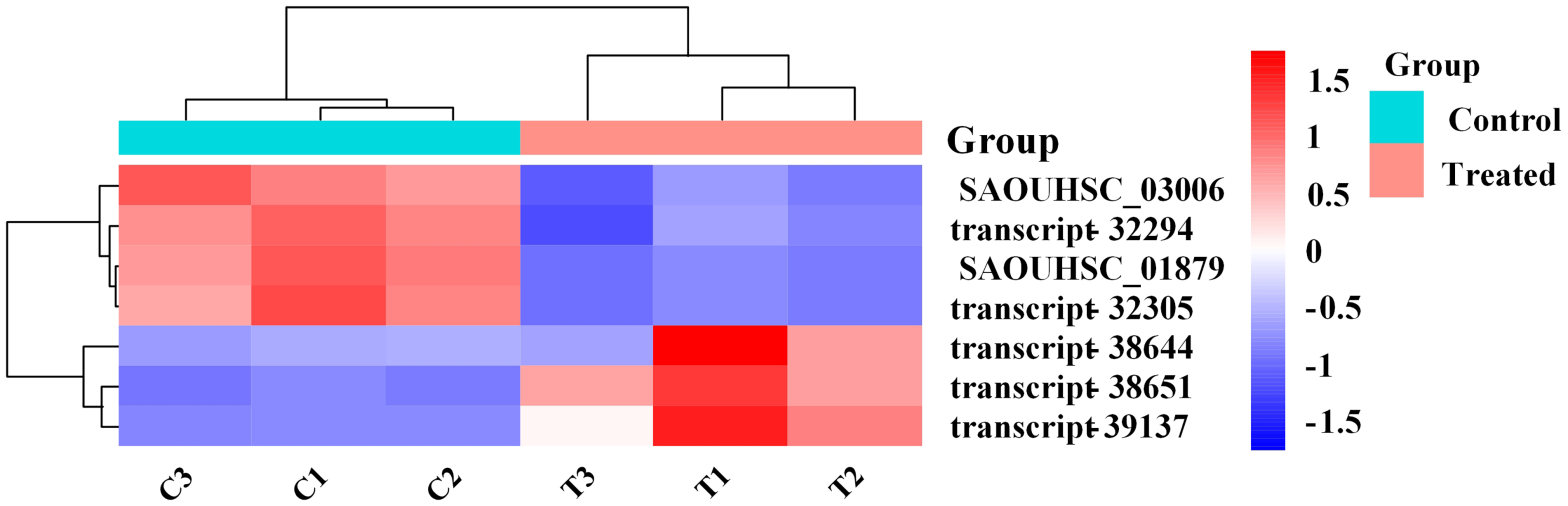
Validation of RNA-Seq expression for selected genes by RT-qPCR.

Finding alterations in biological functions is crucial in determining how the antimicrobial activity works. Previous studies showed that the antimicrobial activity of plant flavonoids was closely related to their membrane interactive properties (Zheng et al., 2019; Wang, J. et al., 2020; Yuan et al., 2021). Indeed, the results of this study have also proved that FCS has an inhibition effect on *S. aureus* by destroying the bacterial cell wall and cytoplasmic membrane. Based on this fact, DEGs associated with this mechanism of antibacterial effect were explored in detail (Supplementary Table S3). As shown in Figure 6, genes encoding the two-component regulatory system (TCRS; ko02020), *vraS* and *vraR* (*vraSR*), were upregulated by FCS 2.7-fold and 3.0-fold change, respectively. This system could increase the expression of genes related to peptidoglycan biosynthesis, thereby reducing the damage to the bacteria wall or cytoplasmic membrane (McCallum et al., 2011). Peptidoglycan plays an important role in the bacterial cell wall and that peptidoglycan biosynthesis is essential to cell division, which is also considered a target of antimicrobial agents (Brown et al., 2015). We found the expression levels of *murA*, and *sgtA/B* genes involved in peptidoglycan biosynthesis (ko00550) were up-regulated 1.4-fold and 3.7-fold change, respectively, upon the treatment to FCS. In *S. aureus*, the *murA* and *murZ* both encode UDP-nacetylglucosamine enolpyruvyl transferase, which catalyzes the first committed step in peptidoglycan biosynthesis and therefore plays an important role in cell wall growth (Du et al., 2000). Similarly, Wen et al. (2022) have proved that the MurA and MurC genes of *L. monocytogenes*, which are associated with peptidoglycan biosynthesis, were significantly downregulated in the group treated with the moringin from *Moringa oleifera* seeds. Additionally, the gene encoding *mrcA* located in the final stage of peptidoglycan biosynthesis was up-regulated 2.2-fold change after FCS treatment, which also indicated that peptidoglycan biosynthesis may be induced. The cell wall of Gram-positive bacteria protects cells from environmental stress tolerance, antibiotic susceptibility, host immune evasion, and overall virulence (Kovacs et al., 2019). As with the transcriptome results, TEM revealed that FCS treatment inhibited *S. aureus* by disrupting the structure of the cell wall (Figure 3B). Combined with the research of Utaida et al. (2003), it is speculated that cell wall synthesis in *S. aureus* was induced to repair damaged or missing cell walls, avoid the effects of the extracellular environment and the excessive escaping of intracellular components (Figure 7).

Another underlying antibacterial mechanism of FCS may be associated with the effect of DNA, which is an important part of the cytoplasm (Ning et al., 2017). Hence, DNA replication and repair systems are also essential in bacterial normal physiological activities. Compared to the control, many genes involved in this system were significantly up-regulated after being treated with FCS, including *ssb* for DNA replication (ko03030), *CAD*, *UMPS*, *ndk*, *punA*, *psuG*, and *psuK* for pyrimidine metabolism (ko00240), *adk*, *relA*, *ndk*, *punA*, and *arcC* for purine metabolism (ko00230). Thus, it implies that DNA synthesis would be inhibited when cells were exposed to FCS, and the expression levels of genes associated with DNA replication and repair systems were increased to protect cells from the corresponding stress response. In agreement with our study, the study by Yang et al. (2020). also found the DNA bands with *Larimichthys crocea* whey acidic protein-derived peptide (LCWAP) treatment were darkened or disappeared, indicating that LCWAP could damage the genomic DNA of *S. aureus*, with the damage positively correlated with the concentration of LCWAP.

As we can observe from the bioinformatic analysis, the pathways of differential gene enrichment were ABC transporter (ko02010) and ribosome (ko03010). ABC transporter (ATP binding cassette transporters) are a large class of transmembrane transporters on the cell membrane, which can help bacteria to utilize helpful substances and discharge harmful substances (Hiron et al., 2007). FCS can up-regulate oligosaccharide, polyol and lipid transporters, and monosaccharide transporters, while down-regulate phosphate and amino acid transporters, peptide and nickel transporters. In addition, compared with the control group, the ribosomal coding related genes of *S. aureus* treated with FCS were significantly up-regulated, such as L5, L6, L18, L30, L15 of 50S ribosomal proteins, S8, S5, S9, S15, S18 of 30S ribosomal proteins and so on. The results showed that FCS may play an inhibitory role by inhibiting the function of ribosome components and interfering with the synthesis of bacterial proteins.

In addition, we observed that the expression levels of *ClfA*, *SCIN*, *Hlg* and *Luc* involved in *S. aureus* infection (ko05150) were up-regulated 2.1-fold, 2.7-fold, 2.2-fold and 3.9-fold with the treatment of FCS, respectively. Clumping factor A (*ClfA*) is the major fibrinogen-binding protein on the surface of cells from the stationary phase of growth, which has been reported to act as a protective antigen and a virulence determinant (Josefsson et al., 2001). The up-regulation of *clfA* gene implied the protection of *S. aureus* by *clfA*. Furthermore, the synthesis of ATP by oxidative phosphorylation (ko00190) is a complex membrane-associated process that generates cellular energy in eukaryotes and many prokaryotes. We observed that the expression level of *alpha* and *epsilon* that encoded the components of the F-type ATPase were both up-regulated 2.0-fold after the treatment. Further, genes *QoxC* and *QoxD* encoding cytochrome c oxidase subunits were up-regulated 2.3-fold and 2.6-fold, respectively. This observation indicated FCS has effects on energy metabolism in bacterial cells. The results were consistent with those of Bai et al. (2022), who proved that shikimic acid (SA) and quinic acid (QA) play inhibitory effects on *S. aureus* by influencing oxidative phosphorylation, membrane fluidity, protein synthesis and so on by combined transcriptomic and metabolomic analyses. Overall, the antibacterial action of FCS may act in multiple ways and the complete mechanism has yet to be fully elucidated. In future research, the verification of the key genes and the related functions should be further evaluated and the molecular mechanisms of FCS against *S. aureus* should be explored in depth.

In this study, we provide some novel insights into the molecular mechanism responsible for the antibacterial activity of FCS against *S. aureus*. The results showed that FCS has potential inhibition effects on tested strains that can destroy the bacterial cell wall and membrane, reveal intracellular components, and cause DNA damage. The transcriptome analysis showed that DEGs of *S. aureus* with FCS treatment were mostly involved in the biosynthesis of bacteria wall and membrane and DNA replication and repair, which further demonstrated the antibacterial mechanism of FCS to *S. aureus*. Conclusively, the above results may provide a promising method for the application of *C. salicifolius* S. Y. Hu. and a guide for the exploitation and application of this flavonoid as an antimicrobial agent in the food industry.

## Data availability statement

The datasets presented in this study can be found in the NCBI Sequence Read Archive (https://www.ncbi.nlm.nih.gov/bioproject) under accession number PRJNA911857. Raw reads of the RNA-seq from six samples were deposited in the NCBI SRA (http://www.ncbi.nlm.nih.gov/Traces/sra) under accession number SRR22727864‐ SRR22727869.

## Author contributions

HZ, LC, and WW conceived and designed the experiments. HZ, KO, and QZ analyzed the data. HZ wrote the draft manuscript. HZ and WW revised the manuscript. All authors have read and approved the final manuscript for submission.

## Funding

The authors gratefully acknowledge the financial supports by National Natural Science Foundation of China (No. 31560459), Major Discipline Academic and Technical Leaders Training Program of Jiangxi Province (Grant No. 20182BCB22003), the Earmarked Fund for Jiangxi Agriculture Research System (JXARS-13), and the Graduate Innovative Special Fund Projects of Jiangxi Agricultural University (Grant No. NDYC2020-S013).

## Conflict of interest

The authors declare that the research was conducted in the absence of any commercial or financial relationships that could be construed as a potential conflict of interest.

## Publisher’s note

All claims expressed in this article are solely those of the authors and do not necessarily represent those of their affiliated organizations, or those of the publisher, the editors and the reviewers. Any product that may be evaluated in this article, or claim that may be made by its manufacturer, is not guaranteed or endorsed by the publisher.
